# Association of Airway Disease and Single-Nucleotide Variants in *PTPN14* and *IL9RP3* in Japanese Cases of Rheumatoid Arthritis

**DOI:** 10.3390/jpm16100498

**Published:** 2026-09-25

**Authors:** Takashi Higuchi, Shomi Oka, Kota Shimada, Shinichiro Tsunoda, Satoshi Ito, Misuzu Fujimori, Tadashi Nakamura, Michita Suzuki, Koichiro Saisho, Satoshi Shinohara, Toshihiro Matsui, Kiyoshi Migita, Shouhei Nagaoka, Shigeto Tohma, Kenji Itoh, Hiroshi Furukawa

**Affiliations:** 1 Department of Rheumatology, NHO Tokyo National Hospital, 3-1-1 Takeoka, Kiyose 204-8585, Japan; takashi.qef@ac.auone-net.jp (T.H.); oka-tkb@umin.org (S.O.); shige1010ma@outlook.jp (S.T.); kenji-tky@umin.ac.jp (K.I.); 2 Clinical Research Center for Allergy and Rheumatology, NHO Sagamihara National Hospital, 18-1 Sakuradai, Minami-ku, Sagamihara 252-0392, Japan; ninja02matsui@gmail.com; 3 Department of Rheumatology, NHO Sagamihara National Hospital, 18-1 Sakuradai, Minami-ku, Sagamihara 252-0392, Japan; kouta_shimada@tmhp.jp; 4 Department of Rheumatic Diseases, Tokyo Metropolitan Tama Medical Center, 2-8-29 Musashi-dai, Fuchu 183-8524, Japan; 5 Division of Rheumatology, Department of Internal Medicine, Hyogo College of Medicine, 1-1 Mukogawa-cho, Nishinomiya 663-8501, Japan; shinrintron@gmail.com; 6 Department of Nephrology, Sumitomo Hospital, 5-3-20 Nakanoshima, Kita-ku, Osaka 530-0005, Japan; 7 Department of Rheumatology, Niigata Rheumatic Center, 1-2-8 Hon-cho, Shibata 957-0054, Japan; s-ito@water.ocn.ne.jp; 8 Department of Rheumatology, NHO Himeji Medical Center, 68 Hon-machi, Himeji 670-8520, Japan; misuzufujimori@yahoo.co.jp; 9 Department of Rheumatology, Sakurajyuji Hospital, 1-1-1 Miyukikibe, Minami-ku, Kumamoto 861-4173, Japan; t1.nakamura@sakurajyuji.jp; 10 Department of Internal Medicine, NHO Nagoya Medical Center, 4-1-1 Sannomaru, Naka-ku, Nagoya 460-0001, Japan; suzuki.michita.cb@mail.hosp.go.jp; 11 Department of Orthopedics/Rheumatology, NHO Miyakonojo Medical Center, 5033-1 Iwayoshi-cho, Miyakonojo 885-0014, Japan; saisho889@miyazaki-catv.ne.jp; 12 Tanimura Hospital, 10-2 Kitakoji, Nobeoka 882-0041, Japan; 13 Tochigi Rheumatology Clinic, 1-1-9 Ekimaedori, Utsunomiya 321-0964, Japan; shinohara-trc@nifty.com; 14 Clinical Research Center, NHO Nagasaki Medical Center, 2-1001-1 Kubara, Omura 856-8562, Japan; k-migita@sfh.or.jp; 15 Department of Gastroenterology and Rheumatology, Fukushima Medical University School of Medicine, 1 Hikarigaoka, Fukushima 960-1295, Japan; 16 Department of Internal Medicine, St. Francis Hospital, 9-20 Komine-machi, Nagasaki 852-8125, Japan; 17 Department of Rheumatology, Yokohama Minami Kyosai Hospital, 1-21-1 Rokuura-Higashi, Kanazawa-ku, Yokohama 236-0037, Japan; nagaokascrt@msn.com

**Keywords:** rheumatoid arthritis, airway disease, single-nucleotide variant, genetic association

## Abstract

**Objectives:** Airway disease, commonly related to rheumatoid arthritis (RA), confers an inferior prognosis. Few genetic analyses in RA have investigated patient susceptibility to airway disease. We examined if single-nucleotide variants related to idiopathic pulmonary fibrosis susceptibility were related to airway disease in Japanese cases of RA. **Methods:** Genotyping for rs4233306 [C/T] in *PTPN14* and rs3930589 [T/C] in *IL9RP3* in 246 patients with RA and airway disease, and 425 with RA but without chronic lung disease was performed. **Results:** *PTPN14* rs4233306C and *IL9RP3* rs3930589T were significantly related to airway disease in RA under a dominant model for minor alleles (*p* = 0.0398; odds ratio [OR] 1.41; 95% confidence interval [CI] 1.02–1.96; *p* = 0.0462; OR 3.19; 95% CI 1.06–9.63, respectively). Stratification by male sex resulted in the remaining significant association of *PTPN14* rs4233306 with airway disease in RA cases (*p* = 0.0028; OR 3.70; 95% CI 1.55–8.84). A significant relationship between *IL9RP3* rs3930589 and airway disease in RA patients aged > 65 years was noted (*p* = 0.0135; OR 9.79; 95% CI 1.21–79.20). **Conclusions:** This is the first study to report associations of rs4233306 and rs3930589 with airway disease in Japanese RA cases.

## 1. Introduction

Recently, treatment of rheumatoid arthritis (RA), where synovial joint destruction is caused by autoimmunity, has improved [1]. RA is complicated with extra-articular manifestations including interstitial lung disease (ILD), airway disease, emphysema, or pleuritis [2,3]. Patients with RA frequently have complications related to airway disease [4,5,6,7], and RA causes airway disease [8,9]. Airway disease confers a poor prognosis to RA patients [10,11], and therefore, the elucidation of airway disease pathogenesis in RA is urgently required.

RA pathogenesis mechanisms are currently unclear. Susceptibility to RA and its complications might be related to genetic and/or environmental factors. A single-nucleotide variant (SNV) in *MUC5B* is related to ILD in patients with RA [12,13], and SNVs in *FAM13A*, *TOLLIP*, *TERT,* and *MUC5B* were linked to ILD in European patients with RA [14]. Few genetic studies of ILD in RA have investigated patient genetic susceptibility to airway disease [5,15,16,17,18,19,20].

Previous studies indicated *PTPN14* and *IL9RP3* were associated with idiopathic pulmonary fibrosis (IPF) [21,22,23]. *PTPN14* and *IL9RP3* are involved in pathways relevant to lung remodeling and immune signaling. Because prior work suggests partial overlap in genetic susceptibility between ILD and airway disease [14,20,24], we evaluated whether SNVs in these genes are associated with airway disease in Japanese RA patients.

## 2. Materials and Methods

### 2.1. Patients

Native Japanese living in Japan fulfilling the Rheumatoid Arthritis Classification Criteria [25] or American College of Rheumatology Criteria for RA [26] were recruited to this study from 2010 to 2024. High-resolution or conventional computed tomography (CT) chest images of RA patients performed by hospitals containing research laboratories organized by the NHO Tokyo National Hospital were used to diagnose RA with AD or lack of chronic lung disease (CLD) [19]. Airway disease was defined on chest CT as bronchiectasis (bronchial dilatation, bronchial wall thickening, or atelectasis) or bronchiolitis (centrilobular or peribronchial nodules and branching linear structures). Patients with radiographic ILD were classified separately and were not included in the airway disease group analyzed here. CLD (−) patients had no detectable lung abnormalities on high-resolution or conventional chest CT. Two hundred and forty-six RA patients with airway disease and 425 lacking CLD were enlisted (Table 1).

Allele frequencies of *PTPN14* rs4233306 and *IL9RP3* rs3930589 in the Japanese general population were gained from the 60KJPN Japanese Multi Omics Reference Panel (https://jmorp.megabank.tohoku.ac.jp/202206 (accessed on 8 May 2025)) [27].

The present study was reviewed and approved by the Research Ethics Committees of NHO Sagamihara National Hospital (approval code: 21), NHO Tokyo National Hospital (approval code: 190010) and other organizations involved in this research. Written informed consent was gained from all study participants. This analysis was performed according to the Helsinki Declaration.

### 2.2. Genotyping

Genotyping for rs4233306 [C/T] in *PTPN14* and rs3930589 [T/C] in *IL9RP3* was conducted using TaqMan assays (Assay ID: C__27891898_10 and C__27501388_10, respectively; Thermo Fisher Scientific Inc., Waltham, MA, USA) on a 7500 Fast Real-Time PCR System (Thermo Fisher Scientific Inc.). Thermal cycling settings were: denaturation (95 °C—20 s), 40 cycles (95 °C—3 s), and final step (60 °C—30 s). Expression quantitative trait locus (eQTL) data were examined using the GTEx portal (https://gtexportal.org/home) to explore potential functional effects of the studied SNVs.

### 2.3. Statistical Analyses

Any relationships between variants when comparing RA with no CLD were determined via Fisher’s exact test with 2 × 2 contingency tables under the dominant model for minor alleles. Statistical analyses were performed using BellCurve for Excel (SSRI Co., Ltd., Tokyo, Japan). Stratified analyses were conducted in patients aged >65 years or those who were male. Eighty percent statistical power was attained when the odds ratio (OR) was ≥1.63 for rs4233306 and 4.72 for rs3930589 [28]. A statistically significant finding was shown when *p* < 0.05.

## 3. Results

### 3.1. Characteristics of RA Patients

Table 1 shows the clinical characteristics of individuals with RA with airway disease or without CLD. Those with airway disease had higher age and age at onset compared with those without CLD. RA patients with airway disease had a lesser body mass index. Positivity rates of rheumatoid factor and anti-citrullinated peptide antibody were higher in RA with airway disease. Thus, clinical manifestations were skewed in RA with airway disease.

### 3.2. Association of PTPN14 rs4233306 and IL9RP3 rs3930589 with Airway Disease in RA

*PTPN14* rs4233306 and *IL9RP3* rs3930589 genotyping was performed using RA patient samples (Table 2). A deviation from Hardy–Weinberg equilibrium was detected for *IL9RP3* rs3930589 (*p* = 0.0266) but not *PTPN14* rs4233306 (*p* = 0.7660). Whether *PTPN14* rs4233306 was related to airway disease in Japanese RA cases was studied (Table 2). *PTPN14* rs4233306C and *IL9RP3* rs3930589T were significantly related to airway disease in RA under a dominant model for minor alleles (*p* = 0.0398; odds ratio [OR] 1.41; 95% confidence interval [CI] 1.02–1.96; *p* = 0.0462; OR 3.19; 95% CI 1.06–9.63, respectively) indicating an association between *PTPN14* rs4233306 and *IL9RP3* rs3930589 with airway disease in Japanese RA patients.

### 3.3. Association of PTPN14 rs4233306 and IL9RP3 rs3930589 with Airway Disease in RA Subpopulations

The relationship of *PTPN14* rs4233306 with airway disease was analyzed in patients aged >65 years or those who were male (Table 3) to exclude differences in age and sex between the two groups. Stratification by male sex resulted in the remaining significant association of *PTPN14* rs4233306 with airway disease in RA cases (*p* = 0.0028; OR 3.70; 95% CI 1.55–8.84), and there was a significant relationship between *IL9RP3* rs3930589 and airway disease in RA cases aged >65 years (*p* = 0.0135; OR 9.79; 95% CI 1.21–79.20). Thus, significant associations were observed for *PTPN14* rs4233306 with airway disease in RA males and *IL9RP3* rs3930589 with RA patients aged >65 years.

### 3.4. Association of PTPN14 rs4233306 and IL9RP3 rs3930589 with RA

We also investigated whether *PTPN14* rs4233306 and *IL9RP3* rs3930589 were related to RA per se (Supplementary Table S1). However, no associations between SNVs and RA were detected.

## 4. Discussion

*PTPN14* rs4233306C and *IL9RP3* rs3930589T were revealed as risk alleles for airway disease in Japanese RA patients, although *PTPN14* rs4233306 and *IL9RP3* rs3930589 were associated with ILD in European populations [21,22]. Therefore, the novel finding in this analysis is a relationship amongst these SNVs and airway disease in RA patients.

*PTPN14* rs4233306C is a known risk allele for IPF [21,22]. Notably, we report that *PTPN14* rs4233306C was related to airway disease in RA patients, especially males. This risk allele was related to lower levels of *PTPN14* gene activation in brains according to the Expression Portal Database (*p* = 1.14 × 10^−4^; Supplementary Figure S1A; https://gtexportal.org/home/snp/rs4233306 (accessed on 8 May 2025)) [29], but not with a reduced telomere length in leukocytes [21]. However, this observation derives from non-pulmonary tissues and does not by itself demonstrate altered expression in lung or airway cells. The *PTPN14* gene, expressed in fibroblasts, encodes *PTPN14*, a signaling molecule of the protein tyrosine phosphatase family that regulates cell growth. *PTPN14* deficiency causes choanal atresia and lymphedema, and lymphatic hyperplasia with lymphedema was observed in *PTPN14* mutant mice [30]. Overexpression of *PTPN14* protein was observed in fibroblast-like synoviocytes in RA cases compared with osteoarthritis cases [31]. Thus, the *PTPN14* rs4233306C allele might be involved in the susceptibility to airway disease in RA.

A previous study indicated that *IL9RP3* rs367849850del was primarily related to IPF in European populations, although an association with *IL9RP3* rs3930589 was also observed [21]. Because the allele frequency of *IL9RP3* rs367849850del is quite low in Japanese populations (minor allele frequency = 0.000025; 60KJPN Japanese Multi Omics Reference Panel) (https://jmorp.megabank.tohoku.ac.jp/202206/ (accessed on 8 May 2025)) [27], it is difficult to estimate the genetic effects of *IL9RP3* rs367849850del on the susceptibility of airway disease in RA with this sample size. Thus, *IL9RP3* rs3930589 was selected for analysis in the current study. We found that *IL9RP3* rs3930589 was related to airway disease in RA, especially in older patients with RA. Because of the relatively low allele frequency of *IL9RP3* rs367849850del, *IL9RP3* rs3930589T might be the main effector SNV for the development of airway disease in Japanese RA. The *IL9RP3* rs3930589T risk allele was related to lower expression levels of the *IL9RP3* pseudogene or *WASIR2* antisense in the spleen according to the expression portal database (*p* = 4.42 × 10^−17^; *p* = 6.78 × 10^−14^, respectively; Supplementary Figure S1B,C; https://gtexportal.org/home/snp/rs3930589 (accessed on 8 May 2025)) [29]. *IL9RP3* rs3930589T might influence the expression of the *IL9R* gene via *WASIR2* antisense and modulate the activation of signal transducer and activator of transcription and Janus kinase proteins. Thus, the *IL9RP3* rs3930589T allele might be involved in the susceptibility to airway disease in RA patients.

## 5. Conclusions

Our novel finding was the association between *PTPN14* rs4233306C and *IL9RP3* rs3930589T with airway disease in RA patients. Limitations of this analysis included a modest sample size consisting of Japanese populations only. It will be necessary to accomplish large-scale, multi-ethnic replication analyses to elucidate the pathogenesis of airway disease in RA cases. Although stratified analyses were performed in this study, multiple logistic regression analyses should be conducted to eliminate the effects of different clinical characteristics of airway disease in RA patients compared with RA lacking CLD. In conclusion, we demonstrated a relationship between *PTPN14* rs4233306C and *IL9RP3* rs3930589T with airway disease in RA patients, highlighting their potential genetic roles in the pathogenesis of airway disease in RA.

## Figures and Tables

**Table 1:** Characteristics of RA patients in this study.

Clinical Manifestations	Airway Disease	CLD (−)	*p*
Number	246	425	
Male, n (%)	49 (19.9)	66 (15.5)	* 0.1669
Mean age, years (SD)	67.4 (11.4)	61.6 (12.7)	5.78 × 10^−9^
Age at onset, years (SD)	54.3 (15.0)	51.1 (14.8)	0.0410
Steinbrocker stage 3, 4, n (%)	109 (52.7)	63 (37.1)	* 0.0026
Ever smoker, n (%)	66 (65.1)	64 (56.8)	* 0.1427
BMI, kg/m^2^ (SD)	21.0 (3.5)	22.1 (3.6)	0.0047
RF positive, n (%)	193 (88.5)	222 (78.2)	* 0.0028
ACPA positive, n (%)	192 (86.9)	202 (77.7)	* 0.0091

RA: rheumatoid arthritis; CLD: chronic lung disease; RF: rheumatoid factor; BMI: body mass index; ACPA: anti-citrullinated peptide antibody. Data are presented as the mean value or number of each group. Phenotype frequencies or standard deviations are shown in parenthesis. Differences were tested by *t*-test or Fisher’s exact test using 2 × 2 contingency tables. * Fisher’s exact test was used.

**Table 2:** Genotype frequencies of *PTPN14* rs4233306 and *IL9RP3* rs3930589 in RA patients in this study.

*PTPN14*		Genotype			Dominant model for minor alleles		
rs4233306	n	[C/C]	[T/C]	[T/T]	*p*	OR	95% CI
airway disease (+) RA, n (%)	246	164 (66.7)	72 (29.3)	10 (4.1)	0.0398	1.41	(1.02–1.96)
CLD (−) RA, n (%)	425	249 (58.6)	156 (36.7)	20 (4.7)			
*IL9RP3*		Genotype			Dominant model for minor alleles		
rs3930589	n	[T/T]	[T/C]	[C/C]	*p*	OR	95% CI
airway disease (+) RA, n (%)	246	0 (0.0)	9 (3.7)	237 (96.3)	0.0462	3.19	(1.06–9.63)
CLD (−) RA, n (%)	425	1 (0.2)	4 (0.9)	420 (98.8)			

RA: rheumatoid arthritis; CLD: chronic lung disease; OR: odds ratio; CI: confidence interval. Genotype frequencies are shown in parentheses (%). Associations were tested by Fisher’s exact test using 2 × 2 contingency tables under the dominant model for minor alleles.

**Table 3:** Genotype frequencies of *PTPN14* rs4233306 and *IL9RP3* rs3930589 in subpopulations of RA patients in this study.

rs4233306		Genotype			Dominant model for minor alleles		
Age > 65	n	[C/C]	[T/C]	[T/T]	*p*	OR	95% CI
airway disease (+) RA, n (%)	151	104 (68.9)	39 (25.8)	8 (5.3)	0.1072	1.46	(0.92–2.31)
CLD (−) RA, n (%)	176	106 (60.2)	64 (36.4)	6 (3.4)			
Male		[C/C]	[T/C]	[T/T]	*p*	OR	95%CI
airway disease (+) RA, n (%)	49	40 (81.6)	8 (16.3)	1 (2.0)	0.0028	3.70	(1.55–8.84)
CLD (−) RA, n (%)	66	36 (54.5)	25 (37.9)	5 (7.6)			
rs3930589		Genotype			Dominant model for minor alleles		
Age > 65	n	[T/T]	[T/C]	[C/C]	*p*	OR	95% CI
airway disease (+) RA, n (%)	151	0 (0.0)	8 (5.3)	143 (94.7)	0.0135	9.79	(1.21–79.20)
CLD (−) RA, n (%)	176	0 (0.0)	1 (0.6)	175 (99.4)			
Male		[T/T]	[T/C]	[C/C]	*p*	OR	95% CI
airway disease (+) RA, n (%)	49	0 (0.0)	2 (4.1)	47 (95.9)	0.1794	7.00	(0.33–149.17)
CLD (−) RA, n (%)	66	0 (0.0)	0 (0.0)	66 (100.0)			

RA: rheumatoid arthritis; CLD: chronic lung disease; OR: odds ratio; CI: confidence interval. Genotype frequencies are shown in parentheses (%). Associations were tested by Fisher’s exact test using 2 × 2 contingency tables under the dominant model for minor alleles.

## Data Availability

Data supporting the study findings are presented in this paper and supplementary data. Other data are obtainable from the authors upon reasonable request. Clinical and genotype data for each study participant are not available under the conditions of informed consent mandated by the Act on the Protection of Personal Information.

## Supplementary Materials

The following supporting information can be downloaded at https://www.mdpi.com/article/10.3390/jpm16100498/s1, Figure S1: Associations of SNVs with gene expression levels. Table S1: Allele frequencies of rs4233306 and rs3930589 in the RA patients and controls.

## Abbreviations

The following abbreviations are used in this manuscript:

CI	confidence interval
CLD	chronic lung disease
CT	computed tomography
ILD	interstitial lung disease
IPF	idiopathic pulmonary fibrosis
OR	odds ratio
RA	rheumatoid arthritis
SNV	single-nucleotide variant
