# Supplementary material for: Association of Airway Disease and Single-Nucleotide Variants in PTPN14 and IL9RP3 in Japanese Cases of Rheumatoid Arthritis
Source: J Pers Med. 2026 Sep 25;16(10):498. doi: 10.3390/jpm16100498 (PMC13706153; doi:10.3390/jpm16100498)
Supplement: Supplementary file 1 [file jpm-16-00498-s001.zip › jpm-4513741-Figure S1.pdf]

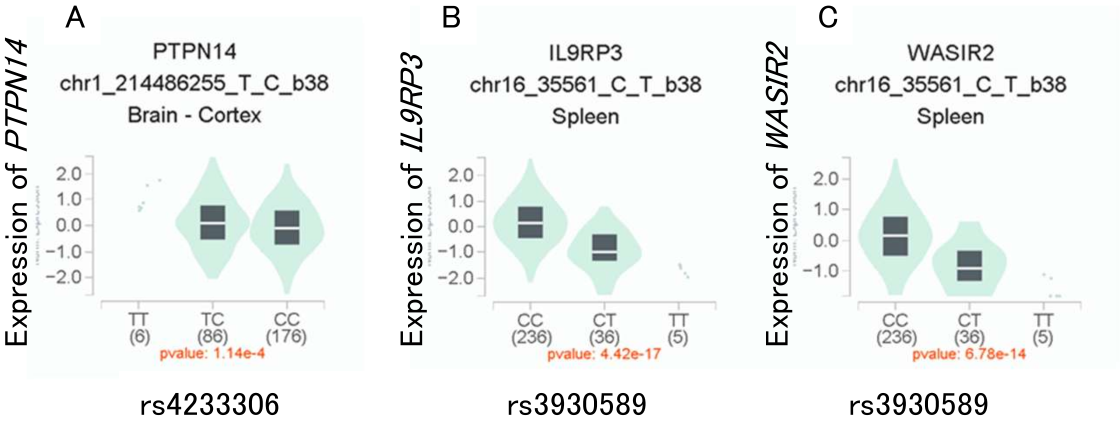

Supplementary Figure S1. Associations of SNVs with gene expression levels. The expression quantitative trait loci analysis in the Genotype-Tissue Expression portal database (https://gtexportal.org/home/snp/rs4233306, https://gtexportal.org/home/snp/rs3930589) showed associations of SNVs with gene expression levels. SNV: single nucleotide variant.
