# Supplementary material for: Association of Airway Disease and Single-Nucleotide Variants in PTPN14 and IL9RP3 in Japanese Cases of Rheumatoid Arthritis
Source: J Pers Med. 2026 Sep 25;16(10):498. doi: 10.3390/jpm16100498 (PMC13706153; doi:10.3390/jpm16100498)
Supplement: Supplementary file 1 [file jpm-16-00498-s001.zip › jpm-4513741-Table S1.pdf]

Supplementary table S1. Allele frequencies of rs4233306 and rs3930589 in the RA patients and controls.

PTPN14		Allele	Allele model		
rs4233306	n	[C]	*P*	OR	95%CI
RA, n (%)	671	1054 (78.5)	0.6196	1.04	(0.91-1.18)
Control, n (%)	59938	93437 (77.9)			
IL9RP3		Allele	Allele model		
rs3930589	n	[T]	*P*	OR	95%CI
RA, n (%)	671	15 (1.1)	1.0000	1.00	(0.60-1.67)
Control, n (%)	59940	1341 (1.1)			

RA: rheumatoid arthritis, OR: odds ratio, CI: confidence interval.
Association was tested in the comparison with the healthy control population by Fisher's exact test using 2x2 contingency tables.
